# Health, financial, and education gains of investing in preventive chemotherapy for schistosomiasis, soil-transmitted helminthiases, and lymphatic filariasis in Madagascar: A modeling study

**DOI:** 10.1371/journal.pntd.0007002

**Published:** 2018-12-27

**Authors:** Jan-Walter De Neve, Rija L. Andriantavison, Kevin Croke, Johannes Krisam, Voahirana H. Rajoela, Rary A. Rakotoarivony, Valérie Rambeloson, Linda Schultz, Jumana Qamruddin, Stéphane Verguet

**Affiliations:** 1 Institute of Global Health, Medical Faculty and University Hospital, Heidelberg University, Heidelberg, Germany; 2 Department of Global Health and Population, Harvard T.H. Chan School of Public Health, Boston MA, United States of America; 3 World Bank Madagascar Country Office, Antananarivo, Madagascar; 4 World Bank, Washington DC NW, Washington, DC, United States of America; 5 Institute of Medical Biometry and Informatics, Heidelberg University, Heidelberg, Germany; Imperial College London, UNITED KINGDOM

## Abstract

**Background:**

Neglected tropical diseases (NTDs) account for a large disease burden in sub-Saharan Africa. While the general cost-effectiveness of NTD interventions to improve health outcomes has been assessed, few studies have also accounted for the financial and education gains of investing in NTD control.

**Methods:**

We built on extended cost-effectiveness analysis (ECEA) methods to assess the health gains (e.g. infections, disability-adjusted life years or DALYs averted), household financial gains (out-of-pocket expenditures averted), and education gains (cases of school absenteeism averted) for five NTD interventions that the government of Madagascar aims to roll out nationally. The five NTDs considered were schistosomiasis, lymphatic filariasis, and three soil-transmitted helminthiases (*Ascaris lumbricoides*, *Trichuris trichiura*, and hookworm infections).

**Results:**

The estimated incremental cost-effectiveness for the roll-out of preventive chemotherapy for all NTDs jointly was USD125 per DALY averted (95% uncertainty range: 65–231), and its benefit-cost ratio could vary between 5 and 31. Our analysis estimated that, per dollar spent, schistosomiasis preventive chemotherapy, in particular, could avert a large number of infections (176,000 infections averted per $100,000 spent), DALYs (2,000 averted per $100,000 spent), and cases of school absenteeism (27,000 school years gained per $100,000 spent).

**Conclusion:**

This analysis incorporates financial and education gains into the economic evaluation of health interventions, and therefore provides information about the efficiency of attainment of three Sustainable Development Goals (SDGs). Our findings reveal how the national scale-up of NTD control in Madagascar can help address health (SDG3), economic (SDG1), and education (SDG4) goals. This study further highlights the potentially large societal benefits of investing in NTD control in low-resource settings.

## Author summary

Neglected tropical diseases (NTDs) cause ill health and disability among the poorest people with extensive economic and social effects. Existing cost-effectiveness analyses, however, have typically focused on the health gains associated with the scale up of preventive chemotherapy. This misses the key fact that health investments can contribute to gains in other sectors leading to broader benefits of NTD control. This study determines the value for money of national NTD control across multiple sectors. We assess the potential health, financial, and education gains of NTD control in a setting where NTDs are highly endemic. We build on extended cost-effectiveness analysis methods to examine five interventions which governments aim to scale up nationally. We find that, per dollar spent, preventive chemotherapy could avert a substantial number of infections, disability-adjusted life years or DALYs, and cases of school absenteeism. Our analysis incorporates broader wealth and education gains into the economic evaluation of health interventions and therefore provides information about the efficiency of attainment of three Sustainable Development Goals. This study highlights the potentially large societal benefits of investing in NTD control.

## Introduction

Neglected tropical diseases (NTDs) affect more than 1 billion individuals worldwide [1]. NTDs are typically a consequence of environmental and socioeconomic conditions and cause ill health and disability among the poorest and most marginalized people [2]. Moreover, the economic and social effects of NTDs are extensive [2, 3]. NTDs affect household and worker productivity [4–6] and are associated with substantial loss of work time [7, 8]. Interventions to control NTDs promise large economic pay-offs outside the health sector in agricultural productivity and educational benefits, and have been considered as investments in human capital and poverty reduction [9]. Given that the existing tools to control NTDs are effective, inexpensive, and the dividends are large, ending these diseases has been suggested as one of the most cost-effective interventions in population health [1]. As a result, a range of elimination and control initiatives for individual diseases has emerged, including the London Declaration on NTDs, inspired by the World Health Organization’s (WHO) 2020 roadmap on NTDs, and the Sustainable Development Goal (SDG) target 3.3 to end the epidemic of NTDs by 2030 [10].

Existing cost-effectiveness analyses of NTD control, however, have typically focused on the health gains associated with the scale-up of preventive chemotherapy in low-resource settings [1, 2]. This is surprising given the wide array of economic and social benefits of investing in NTD control [11]. The first edition of *Disease Control Priorities* (DCP) already made this point in 1993, further highlighting the broad impact of worm infections (helminthiases) [12]:

“The principal public health significance of the helminthiases resides, then, not in their effect on mortality but, rather, in their consequences of impaired growth and development in children, chronic disability, and long-term impairment of function. These consequences, combined with the extremely high prevalence of many helminthic infections, suggest aggregate outcomes for these conditions that are very substantial from the standpoint of both economic productivity and general welfare.”

The contents of the DCP served as powerful background information for the World Bank’s World Development Report 1993 [13], the estimation of disability-adjusted life years (DALYs), the ‘worm wars’ [14, 15], and the recently published third edition of DCP [16].

Furthermore, given limited resources, policymakers dealing with NTDs face difficult decisions often having to balance multiple sectoral goals beyond health. The typical framework used allocates resources to optimize population health. This approach risks missing the fact that health investments contribute to gains in other sectors and may lead to underestimating the broader benefits of NTD control [17]. If NTD control, for instance, contributes to educational gains, policymakers in the education sector may be willing to contribute to the cost of NTD control in a co-financing approach between the education and health sectors to achieve their respective goals jointly [16, 18]. While previous work has documented the extensive benefits of NTD control across the SDG spectrum [13], our study attempts to comprehensively quantify the extended cost-effectiveness of NTD control across multiple sectors.

To address this objective, we assessed the potential health, financial, and education gains of national NTD control in Madagascar, where NTDs are highly endemic (see **S1 Text** in the Appendix for contextual information). In the spirit of extended cost-effectiveness analysis (ECEA) methods [19, 20], we examine interventions for five NTDs which the government aims to scale-up nationally: “lymphatic filariasis” (LF), “schistosomiasis”, and three intestinal parasites i.e. “soil-transmitted helminthiases” (STH) (*Ascaris lumbricoides*, *Trichuris trichiura*, and hookworm). This study focuses on the cost of scaling up NTD control efforts nationally and its impact on morbidity and mortality, out-of-pocket (OOP) treatment costs for households, as well as on educational outcomes. The goal of this study is not to assess the costs and benefits of NTD elimination in Madagascar; rather we propose a modeling approach and illustrate it by assessing the potential costs and benefits of scaling up NTD control.

## Methods

Building on ECEA, we developed a modeling approach to examine the health gains (infections and DALYs averted), household financial gains (reduction in OOP treatment costs), and education gains (cases of school absenteeism averted, defined in years of schooling gained) resulting from NTD interventions in Madagascar (**Table 1**). These interventions included preventive chemotherapy for schistosomiasis, STH, and LF, delivered through school-based mass drug administration (MDA) [21, 22]. For simplicity, we assumed that these interventions were incremental to the current status quo with no other interventions taking place before their rollout. While this may be the case at the national level, in practice a number of interventions have already occurred at subnational levels. For example, the SECALINE program (“Sécurité alimentaire et nutrition élargie”–or Expanded Food Security and Nutrition) provided school-based deworming in two provinces in Madagascar between 1993 and 2003 [23]. Similarly, there might have been programmatic efforts by scientific institutions (e.g. Institut Pasteur) or non-governmental organizations [22].

**Table 1:** Five neglected tropical diseases (NTD) interventions considered.

	Description	Target population	Number of individuals	Coverage before NTD program (%)	Coverage after NTD program (%)	Source
Schistosomiasis preventive chemotherapy	Provision of praziquantel (600 mg)	5–14 years old	6,325,000	0%	75%	Ministry of Health
Ascariasis preventive chemotherapy	Provision of albendazole (400 mg)	5–14 years old	6,325,000	0%	75%	Ministry of Health
Hookworm disease preventive chemotherapy	Provision of albendazole (400 mg)	5–14 years old	6,325,000	0%	75%	Ministry of Health
Trichuriasis preventive chemotherapy	Provision of albendazole (400 mg)	5–14 years old	6,325,000	0%	75%	Ministry of Health
Lymphatic filariasis preventive chemotherapy	Provision of albendazole (400 mg) and diethylcarbamazine (100 mg)	5–14 years old	6,325,000	0%	72%	Ministry of Health

*Notes*: All five NTD interventions were delivered through mass drug administration. Coverage is defined as therapeutic coverage of the NTD program. Geographic coverage would be 100% after the national scale-up of the NTD program. Our analysis does not examine onchocerciasis or trachoma (other NTDs amenable to preventive chemotherapy) since they are not endemic in Madagascar. Source: Ministry of Health, Madagascar [21, 24, 25].

**Fig 1:**
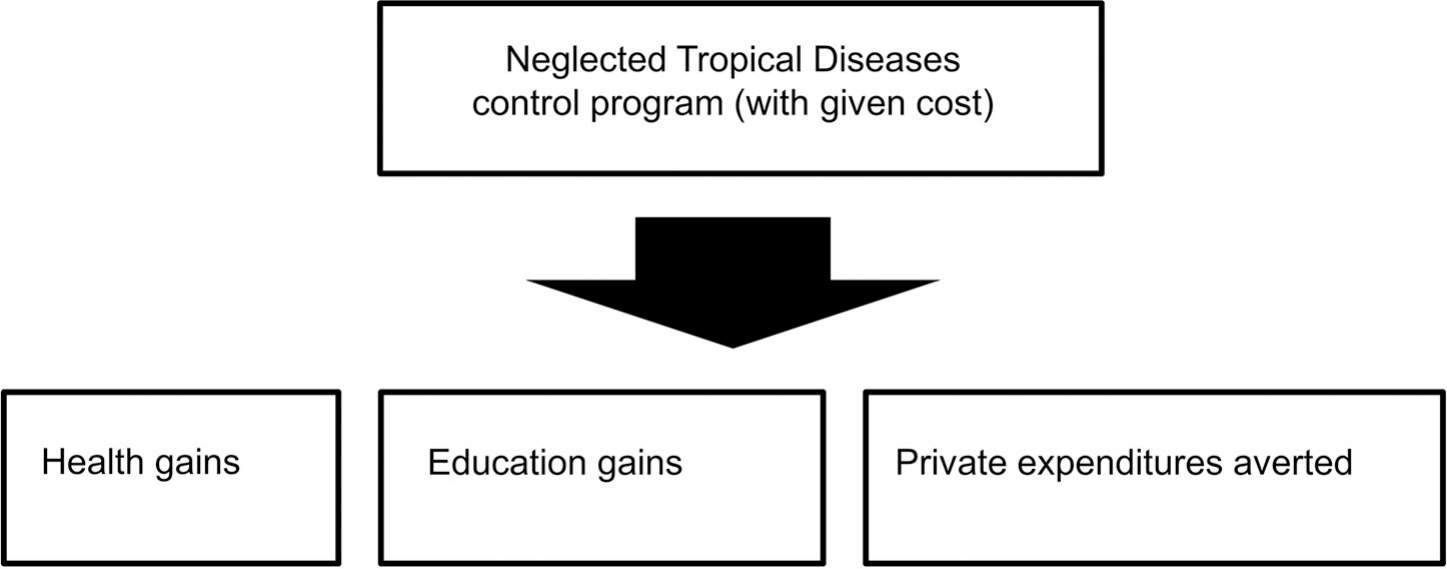
Analytical framework underlying the economic evaluation approach. Analytical structure building on the extended cost-effectiveness analysis methodology where impact is estimated in four domains: cost to the government; health gains (i.e., morbidity and mortality due to infections averted); household financial gains (i.e., private out-of-pocket expenditures averted); and education gains (i.e., increased educational attainment among school-going age children) [19].

**Fig 1** displays an analytical framework for our analysis [19]. For each intervention, we estimated: the number of infections and DALYs averted; the reduction in household financial burden associated with seeking care for NTD-related conditions; and the number of cases of school absenteeism averted (years of schooling gained). These amounts depended on the target population size, NTD prevalence, intervention coverage and effectiveness, the probability of developing clinical conditions resulting from NTD infection, healthcare usage, average household expenditures on NTD treatment, and the education burden assigned to NTD infections. Despite some services being provided free of charge by the government, around 41% of (total) health expenditure are financed privately in Madagascar [26]. Therefore, with free preventive chemotherapy for NTDs, we assumed individuals would still pay on average about 41% of clinical treatment costs for the remaining NTD-related medical issues (e.g., surgical management of hydrocele or lymphedema resulting from chronic LF).

We used government reports to estimate the program costs of the interventions analyzed [21]. **Table 2** displays a breakdown of costs of Madagascar’s 2013 NTD program. Program costs included community mobilization and supervision of staff; whereas all drugs used for the program were donated by pharmaceutical companies. Using the total cost of the campaign and the number of individuals targeted, we inferred the unit cost per individual. In sensitivity analyses, we added drug costs assuming drugs would not be donated by pharmaceutical companies. No other adjustments to the costs data were made. Additional details on Madagascar’s NTD program are provided in **S1 Text** in the Appendix.

**Table 2:** Itemized budget for the neglected tropical disease program in Madagascar (2013 campaign).

*Level*	National (2013 USD)	Regions (2013 USD)	Districts (2013 USD)
Coordination meetings	18,000	-	-
Staff training	32,000	93,000	58,000
Office supplies	13,000	-	-
Tools	36,000	-	-
Community mobilization	16,000	2,000	203,000
Transport	7,000	-	12,000
Supervision	29,000	6,000	243,000
Evaluation meetings	9,000	11,000	84,000
Total	160,000	112,000	600,000

*Notes*: The cost of the campaign was 1,938,962,848 Malagasy Ariary (MGA) (2013) and the number of individuals targeted by the campaign was 4,433,902. Source: Rapport synthétique d'activités et de résultats TMM-PAUSENS 2013 Maladies Tropicales Negligées, Ministère de la Santé Publique, Gouvernement de Madagascar (2013).

We selected input parameters for our economic model in two steps. First, we conducted a narrative literature review of the evidence to identify meaningful estimates for each parameter (Appendix, **S1 Text**). Second, when multiple sources were identified for a parameter, we chose estimates based on source quality and similarity of the context with a Madagascar setting. If source quality or setting was not a differentiating factor, we opted for the more conservative estimate. In additional analyses, we varied our input parameters to assess the sensitivity of our findings to alternative parameter specifications. All parameter inputs used are listed in **Table 3**.

**Table 3:** Summary of the parameters used for modeling of neglected tropical diseases (NTD) program in Madagascar.

	Disease prevalence (%)	Education burden (school years lost)	Health-care use (%)	Household out-of-pocket payments (2013 USD)	Effectiveness of preventive chemotherapy (%)
Schistosomiasis preventive chemotherapy	25% [29]	237,000 [30]	60% [31]	1 [26, 32, 33]	37% [34]
Ascariasis preventive chemotherapy	26% [29]	247,000 [30]	60% [31]	1 [26, 32, 33]	31% [35]
Hookworm disease preventive chemotherapy	7% [29]	66,000 [30]	60% [31]	1 [26, 32, 33]	100% [35]
Trichuriasis preventive chemotherapy	24% [29]	228,000 [30]	60% [31]	1 [26, 32, 33]	13% [35]
Lymphatic filariasis preventive chemotherapy	3% [29]	28,000 [30]	2% [36]	22 [26, 37]	45% [38]

*Notes*: Healthcare use is among those who have clinical symptoms. To calculate household out-of-pocket payments (column 4), we assumed that 41% of (total) health expenditure was financed privately in Madagascar and derived average estimates [26]. We used the effectiveness from mass drug administration campaigns since this was closest to the planned national roll-out of the NTD program in Madagascar. Conversion rates for Malagasy Ariary (MGA) to United States dollar (USD) were 0.00045 (2013) and 0.00055 (2005) using https://www.xe.com/currencycharts/. Inflation from USD 2005 to USD 2013 was taken into account using https://data.bls.gov/cgi-bin/cpicalc.pl (1.0 USD 2005 = 1.2 USD 2013).

We present results for an ‘integrated’ program scenario, where the cost of an NTD campaign targeted to school-going age children covers the roll-out of preventive chemotherapy for all NTDs (as opposed to modeling all five NTD intervention independently–each with its own programmatic cost) [27, 28]. Indeed, the Madagascar NTD program was rolled out together to generate programmatic synergies [21]. Moreover, the same preventive chemotherapy is used to control multiple NTDs. Albendazole, for instance, is used to control all three STH (ascariasis, trichuriasis, and hookworm disease) as well as LF in areas where LF is co-endemic [21].

### Mathematical derivations

We summarize here the mathematical derivations for our key outcomes including: program costs, the number of infections averted, household OOP treatment expenditures averted, and cases of school absenteeism averted (in years of educational attainment gained). For the five NTD interventions (*D* = schistosomiasis, STH, LF), the program targets school-age children (5–14 year-olds, *Pop*_*5-14*_). We denote *p*_*D*_ the probability of treatment for disease *D* conditional on having *D*; *c*_*OOP*,*D*_ the OOP costs for disease treatment; and *c*_*gov*,*D*_ the government costs for disease treatment. Preventive chemotherapy (PC) has an effectiveness *PC*_*eff*_ and the incremental coverage achieved is *Cov*.

From the government perspective, the incremental costs of PC would be:

$$TC_{PC}=\left[\left(n_{doses}*c_{prev}+c_{prog}\right)*Pop_{5-14}\right],$$ (1)

where *n*_*doses*_ is the number of treatment doses; *c*_*prev*_ is the price per dose; and *c*_*prog*_ is the cost of the program per individual. *c*_*prev*_ = 0 when drugs are donated by pharmaceutical companies (we relax this assumption in a sensitivity analysis where we include drug costs), and *n*_*doses*_ = 1 since the campaign included one annual dose.

The number of infections averted is assumed to be:

$$Prev_{post,D}=PC_{eff}*Cov*Prev_{ante,D},$$ (2)

where *Prev*_*ante*,*D*_ is the number of cases of *D* among *Pop*_*5-14*_ before program.

The number of cases of school absenteeism averted would be:

$$School_{post,D}=PC_{eff}*Cov*School_{ante,D},$$ (3)

where *School*_*ante*,*D*_ is the number of cases of school absenteeism associated with NTDs among the school-going age population *Pop*_*5-14*_ before program (i.e., education burden associated with NTD infections). As we identified no data on school years lost due to NTD infections in Madagascar, we used data from a randomized deworming program in primary schools in Kenya to estimate *School*_*ante*_, which found that treatment among children would increase educational attainment by about 0.15 years in the long run [30], and assumed: *School*_*ante*, *D*_ = *Pop*_*5-14*_ * *Prev*_*ante*,*D*_ * 0.15.

The amount of household OOP expenditures averted by the program would be *E*_*post*_ = *Prev*_*post*_ * *p*_*S*_ * *p*_*D*_ * *c*_*OOP*,*D*_, where *Prev*_*post*_ is the number of infections averted, *p*_*S*_ the probability of developing clinical symptoms resulting from *D*, *p*_*D*_ the probability of seeking treatment for *D* (conditional on having *D*), and *c*_*OOP*,*D*_ the OOP costs for *D* treatment.

### Benefit-cost ratio

We examined the ratio of the full spectrum of benefits of NTD control in monetary units divided by the total cost of investing in NTD control. We converted DALYs gained into a monetary value (USD). We assessed a range of monetary values since there is no formal consensus on how much to spend to avert a DALY in low-resource settings [39, 40]. To assess the monetary value of education gains, we used data from the Labor Force Surveys of Madagascar and evidence on the annual rates of return to schooling. The average monthly salary in Madagascar has been estimated at 55,000 Malagasy Ariary (about USD20), or 667,000 Malagasy Ariary (about USD230) per year, whereas the estimated return to one additional year of schooling has been quantified at 11% (of an individual’s wage) [41]. To capture the present value of long-run wage benefits associated with preventive chemotherapy, we conservatively assumed wage benefits over 20 years discounted at 3% per year [42]. We used data on the average wage in Madagascar (as opposed to the minimum wage) since the labor market is dominated by non-wage earners (only 11% of the labor force earns a salary) [43]. The average wage is lower than the minimum wage in the private sector [44].

### Subnational analysis

To assess the distributional impact, we examined the subnational variation in the effects of the program on health, financial, and education gains. Field studies conducted in Madagascar between 2015 and 2016 suggest that the prevalence of STH among school-aged children ranges from 0 to 94% (Appendix, **S1 Fig**). A similarly large range has been observed for schistosomiasis, which ranged from 1 to 89% in endemic districts (106 out of 114 districts). The prevalence of LF ranged from 0 to 58% (in 99/114 districts) [22]. We examined the differential impact of rolling out the program using a range of values for prevalence that is representative of the distributions seen in Madagascar. Specifically, we used 5%, 25%, 45%, 65%, and 85% as prevalence for the five NTDs. For instance, in the districts Ampanihy, Beloha, Ihosy and Toliara II in South-Western Madagascar, the prevalence of STH ranges from 1 to 8%. Conversely, in the districts Mananara Avaratra, Maroantsetra, Toamasina I and Toamasina II in North-Eastern Madagascar, the prevalence of STH ranges from 77 to 99%. A similarly high prevalence of STH is found along the Eastern coast [22]. We expect that the combination of high prevalence and focus on smaller geographic areas increases the cost-effectiveness of NTD control in highly endemic districts. It is likely that national NTD interventions may ultimately focus on specific “hot spots” where NTDs are highly endemic using regionally targeted campaigns.

### Sensitivity analyses

Univariate and multivariate sensitivity analyses were conducted to explore the sensitivity of our findings to various parameter assumptions.

Considerable uncertainty exists around NTDs, including prevalence and intensity of infection and of related conditions, their distributions, effectiveness of MDA to control NTDs, as well as on the effect of NTD control on economic and social outcomes. In particular, there has been some degree of controversy around the effect of NTD control on school participation [14, 15, 45] and previous cost-effectiveness calculations [46]. To acknowledge the uncertainty around our results, we conducted a probabilistic sensitivity analysis using Monte Carlo simulations (n = 1,000) [47, 48], where we assumed selected distributions for each key input parameter including disease prevalence, treatment effectiveness, the percentage of paid workers in Madagascar, healthcare costs, and averted years of school absenteeism (school years gained) (Appendix, **S1 Table**). We then extracted 95% uncertainty ranges (UR) capturing the uncertainty in our findings. We used R statistical software version 3.4.3 for all simulations.

We also examined sensitivity to key input parameters, one at a time. First, we assumed that all drugs were donated by pharmaceutical companies, which may not always be the case, particularly after 2020 (a number of large manufacturers have pledged to continue large medicinal donations under the London Declaration for the period 2014–2020) [49]. We therefore tested alternative input parameters for the drug costs: USD0.080/tablet for schistosomiasis (praziquantel at 2.5 tablets of 600mg per person-year); and USD0.045/tablet (albendazole at 1 tablet of 400mg per person-year) and USD0.004/tablet (diethylcarbamazine (DEC) at 2 tablets of 100mg per person-year) for STH and LF [50] (Appendix, **S2 Table**). Second, we used the total cost of an entire NTD campaign to model each NTD intervention as an independent campaign with its own programmatic costs to further understand the contribution of each NTD subintervention (as opposed to an ‘integrated’ program scenario).

Our main model assumed that targeted individuals were independent of each other. In other words, we assumed that infections averted by preventive chemotherapy for LF infection were unrelated to those infections averted by preventive chemotherapy for STH. In practice, we may be “double-counting” the benefits of NTD interventions in cases where there is co-infection with multiple NTDs within the same individual. This may particularly be the case in contexts where individuals at risk of NTD infection are also those most likely to be co-infected. We therefore studied a conservative scenario where we assumed a “worst-case” scenario of 100% co-infection. In other words, we assumed that all NTD infections co-occurred within the same individuals. (No data was available for Madagascar on the prevalence of co-infection with multiple NTDs within the same individual). To do so, we changed the following input parameters: we took the highest prevalence among NTDs in our target group and assumed that only those individuals were potentially infected with any of the other NTDs—in our application, the highest prevalence found was 26% among school-age children (ascariasis); and we used the lowest effectiveness for preventive chemotherapy among NTD interventions since the prevention of one NTD is insufficient without preventing other NTDs in co-infected individuals (13%, for trichuriasis preventive chemotherapy).

## Results

The extent of health, financial, and education gains varied substantially across the five NTD interventions examined (**Table 4**). The number of infections averted would range from 61,000 (LF) to 439,000 (schistosomiasis). We also present calculations of DALYs averted for each NTD intervention, using estimates on the number of DALYs caused by each NTD from the Global Burden of Disease study (2015) [51]. The number of DALYs averted would range from 300 (trichuriasis) to 4,400 (schistosomiasis). The costs were identical for schistosomiasis, STH, and LF preventive chemotherapy, since drugs were donated by pharmaceutical companies and we examined an ‘integrated’ scenario, where all interventions were covered by a single programmatic delivery platform. Substantial uncertainty accompanied the impact on educational outcomes.

**Table 4:** Infections averted, DALYs averted, household out-of-pocket expenditures averted, and cases of school absenteeism averted for each of the five neglected tropical diseases (NTD) interventions in Madagascar.

	Infections averted (uncertainty intervals)	DALYs averted (uncertainty intervals)	Household expenditure averted in 2013 USD (uncertainty intervals)	Cases of school absenteeism averted in school years (uncertainty intervals)
Schistosomiasis preventive chemotherapy	439,000	4,400	34,000	66,000
	279,000–650,000	2,800–6,500	17,000–65,000	4,000–150,000
Ascariasis preventive chemotherapy	382,000	1,100	30,000	57,000
	250,000–581,000	700–1,700	18,000–49,000	4,000–131,000
Hookworm disease preventive chemotherapy	332,000	1,700	26,000	50,000
	157,000–631,000	800–3,400	10,000–57,000	5,000–141,000
Trichuriasis preventive chemotherapy	148,000	300	12,000	22,000
	79,000–268,000	200–600	5,000–25,000	2,000–59,000
Lymphatic filariasis preventive chemotherapy	61,000	2,400	11,000	9,000
	20,000–177,000	900–6,900	3,000–37,000	1,000–36,000
Total	1,362,000	9,900	113,000	204,000
	785,000–2,307,000	5,400–19,100	53,000–233,000	16,000–517,000

*Notes*: The NTD program was considered to be delivered through mass drug administration nationwide. Uncertainty intervals were constructed using upper and lower bounds for prevalence of NTDs, effectiveness of treatment, DALYs assigned to NTDs, probability of developing NTD-related morbidity and treatment costs in Monte Carlo simulations (n = 1,000 runs) as explained in the Methods section. DALYs per infection used data from the Global Burden of Disease study 2015 (column 2). Link: http://ghdx.healthdata.org/gbd-results-tool.

Schistosomiasis and ascariasis preventive chemotherapy were the two interventions that prevented the highest number of infections per dollar spent, since schistosomiasis chemotherapy prevented 176,000 infections and ascariasis chemotherapy prevented 153,000 infections (per $100,000 spent). Schistosomiasis is highly prevalent (25% nationally) among school-age children and preventive chemotherapy is comparatively effective (at 37%). By contrast, LF preventive chemotherapy averted the smallest number of infections per dollar spent, since the prevalence (3% nationally) is comparatively low.

There was a relatively small reduction in household OOP treatment expenditures. For LF, the program would reduce OOP expenditures for the clinical management of chronic LF (lymphedema and hydrocele) by about $11,000 since healthcare services for chronic LF appeared underutilized in Madagascar (2%) [36]. For schistosomiasis and STH, the program would reduce OOP expenditures for the clinical management of symptoms related to infection by about $12,000 (trichuriasis) to $34,000 (schistosomiasis).

We calculated the incremental cost-effectiveness ratio (ICER) for the entire integrated NTD program by dividing the total program costs by the total number of DALYs averted. The estimated ICER was USD125 per DALY averted (95% UR: 65–231). After converting health and education gains into monetary terms, the benefit-cost ratio was estimated at 13 (5–31) (**Table 5)**. Alternatively, using a monetary value of USD250 per DALY averted, the benefit-cost ratio would become 7 (1–19). These results represent the benefits and costs of investing in an ‘integrated’ NTD program, where the cost of an NTD campaign targeted to school-going age children covers the roll-out of preventive chemotherapy for all five NTDs jointly.

**Table 5:** Infections averted, DALYs averted, household out-of-pocket expenditures averted, and cases of school absenteeism averted in monetary terms for each of the five neglected tropical diseases (NTD) interventions in Madagascar.

	DALYs averted in economic burden (1 DALY = 1,000 USD)	DALYs averted in economic burden (1 DALY = 250 USD)	DALYs averted in economic burden (1 DALY = 2,000 USD)	Household expenditure averted (2013 USD)	Additional wage returns resulting from schooling gains (2013 USD)	Total benefits (2013 USD)
Schistosomiasis preventive chemotherapy	4,350,000	1,088,000	8,700,000	34,000	1,999,000	6,383,000
	2,767,000–6,507,000	692,000–1,627,000	5,534,000–13,014,000	17,000–65,000	120,000–5,659,000	2,796,000–12,231,000
Ascariasis preventive chemotherapy	1,103,000	276,000	2,206,000	30,000	1,726,000	2,859,000
	688,000–1,672,000	172,000–418,000	1,376,000–3,344,000	18,000–49,000	134,000–4,850,000	840,000–6,571,000
Hookworm disease preventive chemotherapy	1,720,000	430,000	3,440,000	26,000	1,514,000	3,260,000
	796,000–3,357,000	199,000–839,000	1,592,000–6,714,000	10,000–57,000	149,000–5,041,000	955,000–8,455,000
Trichuriasis preventive chemotherapy	329,000	82,000	658,000	12,000	666,000	1,007,000
	170,000–596,000	43,000–149,000	340,000–1,192,000	5,000–25,000	55,000–2,247,000	230,000–2,868,000
Lymphatic filariasis preventive chemotherapy	2,423,000	606,000	4,846,000	11,000	273,000	2,707,000
	862,000–6,901,000	216,000–1,725,000	1,724,000–13,802,000	3,000–37,000	20,000–1,321,000	885,000–8,259,000
Total	9,925,000	2,482,000	19,850,000	113,000	6,178,000	16,216,000
	5,283,000–19,033,000	1,322,000–4,758,000	10,566,000–38,066,000	53,000–233,000	478,000–19,118,000	5,706,000–38,384,000

*Notes*: See references for the economic gains associated with one DALY in low-resource settings [39, 40] and additional schooling in Madagascar [41]. We estimated the monetary gains of schooling using data on wages from the Labor Force Survey of Madagascar (2012) [43]. We counted wage benefits over 20 years discounted at 3% per year [42]. The total benefits shown in column 6 are based on a monetary value of 1,000 USD per DALY averted [39].

In **S3 Table** (Appendix), we illustrate the subnational impact by district using a varying prevalence of all NTDs in the integrated NTD program. As expected, in average-sized districts (population of 63,000) with an 85% prevalence of all NTDs (i.e. “hot spots”), preventive chemotherapy would avert a large number of infections, DALYs, and school years lost. In contrast, in districts with a 5% prevalence, preventive chemotherapy would avert a very small number of infections, DALYs, and school years lost. These results imply, as expected, strong geographical variation in the cost-effectiveness of NTD control in Madagascar and the need for regionally targeted approaches.

### Sensitivity analyses

Our results were generally consistent across sensitivity analyses (Appendix, **S4–S6 Tables**). When each NTD intervention was modelled independently with its own programmatic cost (instead of an ‘integrated’ program), the benefit-cost ratio would range from 1 (for trichuriasis preventive chemotherapy) to 5 (for schistosomiasis preventive chemotherapy). When adding drug costs to the campaign costs, the benefit-cost ratio would be reduced to about 4 (**S5 Table**). Taking into account the possibility of co-infection with NTDs within the same individuals (with the conservative 100% overlap), the benefit-cost ratio would be reduced to about 1 (**S6 Table**).

## Discussion

We presented results for a comprehensive economic evaluation of five NTD interventions in Madagascar. We examined the likely consequences of scale-up of NTD preventive chemotherapy in averting infections, in addition to the potential household financial and education gains. This type of analysis distinguishes itself from a traditional cost-effectiveness analysis because it also includes non-health outcomes, allowing policymakers to make comprehensive decisions as they choose between alternative funding strategies given limited resources. The recent institution of the Sustainable Development Goals (SDGs) has garnered increasing attention to jointly improve health and financial protection (SDG3), reduce poverty (SDG1), and increase educational attainment (SDG4). Implementation of complementary investments across sectors is key to reducing and eliminating the NTD burden in the post-2015 development agenda [17]. This study examines the broader spectrum of societal benefits of NTD control.

We modeled each NTD intervention in an ‘integrated’ scenario since NTD interventions are typically rolled out together to generate programmatic synergies [27, 28], including in Madagascar [21]. The cost of an NTD campaign targeted to school-going age children therefore covered the rollout of preventive chemotherapy for all five NTDs. As expected, the benefit-cost ratio increased substantially with integration. For schistosomiasis, for instance, mass treatment would have costed USD286 per DALY if rolled out independently of the other four NTDs. Similarly, NTD interventions may be rolled-out together or in combination with campaigns targeted to other diseases (e.g., immunization or nutrition fortification [2]). The integration of these services could further reduce costs through economies of scope, and produce synergistic effects not captured by our model.

Our cost-effectiveness estimates are somewhat higher than those from previous studies [1]. For instance, a study estimated the cost-effectiveness of school-based mass treatment programs for STH with albendazole or mebendazole at about 2008 USD2-11 per DALY averted [2]. Mass treatment of school-age children in Côte d'Ivoire for STH and schistosomiasis together costed around 2014 USD118 per DALY averted relative to doing nothing [52]. Even though the variances around these estimates may still overlap, there may be a number of reasons for discrepancies between studies [2]. Our modeling, for instance, used effectiveness estimates from mass treatment campaigns in real-world settings that mirrored the scale-up of NTD control in Madagascar [34, 35, 38]; in more controlled settings, the efficacy of preventive chemotherapy for NTDs may be larger [53, 54]. Other possible reasons for discrepancies include alternative choices of disability weights [46] or drugs [2], as well as local social and environmental conditions [55]. A recent review on preventive chemotherapy for STH, however, suggests that most studies present results within the range of being highly cost-effective [56], and NTD interventions seem one of the most cost-effective interventions in public health [1].

Nevertheless, our study presents a number of limitations. First, as with all cost-effectiveness analyses, our findings rely on the availability and quality of the evidence at hand. Limited robust evidence was available for the health, financial, and education burden assigned to NTDs, and the effectiveness of chemotherapy. In our review of the literature, we identified only limited evidence on the long-run education burden due to NTDs (years of schooling lost) [30, 57, 58] and we had no data on disease-specific healthcare usage and OOP spending for four out of five NTDs. Limited data was also available on the incidence of morbidity associated with chronic NTD infection. A recent systematic review on mass deworming, for instance, suggested that certainty in the evidence for long-term effects on educational outcomes was “very low” for STH control [59]. Consistent with the literature, uncertainty intervals around our estimates for cases of school absenteeism averted (in school years gained) were large. There is also controversy around the validity of evidence for schooling outcomes [60]. A trial in Kenya, in which school-based mass treatment with deworming drugs was randomly phased into schools found protective effects of NTD control on school absenteeism [15, 30]. A recent replication study, however, found that deworming may be less effective than previously suggested [14, 45, 61]. Second, we did not capture all benefits or costs of NTD control. For instance, because preventive chemotherapy likely increases labor supply, it could create a fiscal externality through its impact on tax revenues [62]. Conversely, if deworming allows children to go back to school and attain a higher level of schooling, the government may need to invest more in education [30]. Third, the roll-out of NTD control in Madagascar is planned until at least 2020 [22]. The cost-effectiveness of NTD control will likely decrease over time as NTD prevalence reduces. Fourth, due to lack of data, we used disease prevalence although it may not be the best measure to examine interventions addressing macroparasites (as opposed to viral or bacterial infections) [63–65]. The morbidity associated with NTDs is typically experienced by individuals with high parasitemia, hence the impact of an MDA campaign on morbidity would come from reducing the intensity of infections with high parasitemia (rather than prevalence). There may also be positive spillover effects for those not treated due to a reduction in the level of transmission [15, 30]. In our modeling, we did not take into account benefits of curing morbidity in individuals who are still infected but with a lower intensity infection, or possible benefits for those who are not directly treated. These benefits are potentially large as well as long-term (in the case of LF, chronic stages of disease such as hydrocele may be prevented providing health benefits accruing many years after MDA). Our modeling is static rather than dynamic, which would have otherwise captured additional synergies including transmission and indirect effects, but relied on additional data and assumptions around NTDs that is not available. Better data about the life history of NTDs is needed, including intensity of infection, disease severity, and their distributions, as well as transmission and indirect effects [65]. Fifth, the choice of drugs may affect our estimates. The government of Madagascar, for instance, has previously used mebendazole as antihelmintic instead of albendazole in a number of districts [21], which may have fewer benefits due to its lower efficacy against hookworm infection [66]. Sixth, the disability weights for the DALYs are intended to be solely measures of losses of ‘optimal health’, and are not intended to represent losses of economic productivity or well-being [63, 65]. We used the disability weights in the absence of other data on productivity losses due to health, similar to the Copenhagen Consensus exercise [39]. Yet, we focused on school-age children with most long-run economic gains resulting from increased school attainment, which we took into account. Seventh, our analysis focused on the most prevalent NTDs in Madagascar but included little case management of NTDs with high rates of catastrophic expenditure (average OOP treatment expenditures were used). We also did not examine impoverishment (for example, the number of cases of poverty averted). Lastly, our economic evaluation approach is only one method for priority setting. Decision-makers should also consider ethical and political factors, health system constraints, and targeting individuals at high risk (as opposed to the general population).

Economic evaluations for health policy have typically focused on quantifying the health gains per given expenditure. Policymakers in low-resource settings, however, face difficult decisions balancing multiple sectorial goals and require evidence on how to jointly achieve health, economic, and education objectives. This study attempts to comprehensively model the health, financial, and education impact of national NTD control in a resource-limited setting, and contributes to our understanding of how health interventions can affect economic and education aims.

## Supporting information

S1 Text Additional background information and context. (DOCX)

S1 Table Base-case values and uncertainty ranges used in the probabilistic sensitivity analysis. (DOCX)

S2 Table Estimated unit cost of drugs (per tablet) for neglected tropical diseases in Madagascar (2012). (DOCX)

S3 Table Subnational analysis: Cost-effectiveness of neglected tropical disease control by different district prevalence categories in Madagascar. (DOCX)

S4 Table Benefit-cost analysis of neglected tropical disease (NTD) control in Madagascar. Each NTD intervention was modelled independently with its own programmatic cost.
(DOCX)

S5 Table Univariate sensitivity analysis: Drugs used for preventive chemotherapy for neglected tropical diseases in Madagascar are not donated for free by pharmaceutical companies. (DOCX)

S6 Table Benefit-cost analysis of NTD control in Madagascar using an alternative benefit scenario: Assuming all infections occur within the same individuals (100% overlap). (DOCX)

S7 Table Cure rates of selected drugs for control of NTDs. (DOCX)

S1 Fig Prevalence of soil-transmitted helminthiases by district in Madagascar (2016). (DOCX)

S2 Fig Distribution of outpatient visits at primary care centers (“Centres de Santé de Base”) by region in Madagascar (2015). (DOCX)
