# Supplementary material for: Health, financial, and education gains of investing in preventive chemotherapy for schistosomiasis, soil-transmitted helminthiases, and lymphatic filariasis in Madagascar: A modeling study
Source: PLoS Negl Trop Dis. 2018 Dec 27;12(12):e0007002. doi: 10.1371/journal.pntd.0007002 (PMC6307713; doi:10.1371/journal.pntd.0007002)
Supplement: S1 Text — (DOCX) [file pntd.0007002.s001.docx]

## **S1 Text: Additional background information and context**

## **Madagascar study setting**

A large island nation, off the coast of Southeast Africa, Madagascar ranks 158^th^ on the Human Development Index and 158^th^ on the GINI coefficient (GINI of 43) [1]. Gross national income per capita is just US$400 [2], with over 70% of the population living below the national poverty line (1,467 Malagasy Ariary (MGA) or about US$0.50 per day [3]). While life expectancy at birth has increased steadily over time, from 51 years in 1990 to over 65 years in 2015; the total fertility rate remains high at 4.4 births per woman, along with the under-five mortality rate at about 50 per 1,000 live births [4]. Nearly half of the population is aged under 15 years [5]. The epidemiological profile of Madagascar reflects a “double burden of disease”, with the leading causes of death including lower respiratory infections (10% of total annual estimated deaths), cerebrovascular diseases (9%), and diarrheal diseases (8%); and the leading causes of years of life with disability (YLD) being iron-deficiency anemia (9% of total estimated YLDs), sense organ diseases (such as vision loss resulting from cataract) (8%), and depressive disorders (8%) [6]. In 2015, NTDs and malaria constituted about 10% of YLDs [6]. Disability due to NTDs, however, has decreased over the past decade. For instance, the number of YLDs due to schistosomiasis was estimated to have decreased by about 50% between 2005 and 2015 [7]. Structural barriers to health remain a major risk factor for death and disability (i.e. disability-adjusted life year or DALY). The Global Burden of Disease (GBD) study category “unsafe water, sanitation and handwashing”, for instance, was the country’s second most common risk factor for DALYs in 2015 due to diarrhea, lower respiratory infections, and other common infectious diseases [7].

## **Education system**

The public education system in Madagascar is based on the “5-4-3“ system, including five years of primary education (ages 6-10), four years of lower secondary education (ages 11-14), and three years of upper secondary schooling (15-17) [8], in addition to one year of preschool. The *de jure* primary school going age is six years-old, although dropout rates and grade repetition in Madagascar are high. The primary completion rate was 69% in 2014 and repetition rate in primary school (all grades) was 20% in 2014. Education is compulsory for children between the ages of six and fourteen. While gross primary enrollment ratio (both sexes) is high (150% in 2015)^[[1]](#footnote-2)^, the gross lower secondary enrollment ratio (both sexes) was substantially lower at 50%, and the upper secondary enrollment ratio (both sexes) was only 22% in 2014. The “baccalauréat” qualification (the equivalent of a high school diploma) signals the end of upper secondary schooling but few grade 12 students pass the baccalauréat after senior secondary education (only about half in the 2000s [9]). In principle, public primary schooling is free in Madagascar, although households may occur indirect costs (such as books, uniforms, transport to/from school) [10]. Finally, while education data is limited for the country, enrollment rates have been suggested to be relatively similar for women and men; and mean years of schooling has been suggested to be low (5 years among adults [11, 12]).

## **NTD control in Madagascar**

### Prevalence of NTDs

In the context of this study, we examine five NTDs: “lymphatic filariasis” (LF), “schistosomiasis”, and three “soil-transmitted helminthiases” (STHs) including *Ascaris lumbricoides*, *Trichuris trichiura*, and hookworm infections [13-15]. Among school-going age children (ages 5-14), the national prevalence of the five NTDs in Madagascar has been estimated at 25% (schistosomiasis), 3% (LF), 26% (ascariasis), 24% (trichuriasis), and 7% (hookworm disease); whereas among adults (ages 15-49), the prevalence of the five NTDs has been estimated as 40% (schistosomiasis), 5% (LF), 21% (ascariasis), 19% (trichuriasis), and 8% (hookworm disease) [6]. We did not examine other NTDs amenable to preventative chemotherapy such as river blindness and trachoma since the national control program in Madagascar studied in this analysis did not include them [16].

The prevalence of NTDs in Madagascar varies strongly by geographical area. For schistosomiasis, for instance, the prevalence ranges from 1% (in the North and East of Madagascar) to 89% (in the South and West) in endemic districts (106 out of 114 districts are endemic) [17]. A large sentinel survey was conducted in 2015 covering most of the Western half of the country and found a high prevalence and intensity of schistosomiasis in particular. Among children ages 7-10 years of age across 18 districts, the prevalence of *Schistosoma haematobium* and *Schistosoma mansoni* infection was 31% and 5%, respectively^[[2]](#footnote-3)^; whereas the prevalence of any STH infection was 5% [18]. The study found no significant differences in prevalence of infection or heavy-intensity infection between school attending and non-attending children (with the exception of *S. mansoni* infection, which was more common in children who did not attend school regularly). Another recent survey conducted in the South of Madagascar^[[3]](#footnote-4)^ examined the prevalence of LF, schistosomiasis, and STHs [19]. The study found that, among children of school-going age (5-15 years old), the prevalence of LF was 0.1%, prevalence of STHs ranged between 8% (hookworm infection) and 47% (ascariasis), and the prevalence of schistosomiasis was 22%.

In Madagascar, schistosomiasis was linked to 77,000 YLDs (3.0% of total YLDs) and 79,000 DALYs (0.7% of total DALYs) in 2015; whereas LF and STHs were linked to 48,000 (0.4% of total DALYs) and 27,000 DALYs (0.3% of total DALYs), respectively [6]. The case fatality ratio (CFR, defined as the probability of death among cases diagnosed with the disease [20]) was estimated by WHO at about 0.0014% for STHs and schistosomiasis [21]. This estimate, however, appears relatively conservative. Other studies have estimated more than ten-fold higher CFRs, including 0.014% for schistosomiasis [22] and 0.08% for STHs and schistosomiasis [23].

### NTD program roll-out

A few small-scale NTD campaigns were carried out between 2009 and 2012 [19]. In 2013, however, the government of Madagascar, in collaboration with financial and technical partners, initiated a larger NTD control campaign in 24 districts of five regions in the South (Amoron’Imania, Matsiatra Ambony, Vatovavy Fitovinany, Atsimo Atsinanana, Androy). The Ministry of Health aimed to target five endemic NTDs through preventive chemotherapy: schistosomiasis, three major intestinal nematode infections (STHs)—ascariasis, hookworm disease, and trichuriasis—and LF^[[4]](#footnote-5)^. The program was intended to reach 100% geographical coverage (defined as the proportion of administrative units that are implementing preventive chemotherapy of all those that require to be covered) and at least 70% therapeutic coverage (defined as the proportion of individuals requiring preventive chemotherapy who have ingested the appropriate drug) among the age groups 5-14 (schistosomiasis and STHs) and 2+ (LF), by the end of the program within the targeted districts. This is similar to the global target set by WHO in the roadmap on NTDs, which recommends that 75% of pre-school and school-aged children at risk are regularly treated by 2020. The campaign targeted (as opposed to delivered) approximately 4 million individuals for LF treatment (or about 17% of the total population of Madagascar in 2013); including about 1.5 million school-going age children^[[5]](#footnote-6)^ for schistosomiases and STHs treatment. The distribution of drugs was community-based, through either door-to-door visits by community workers or at school for school-going age children. Actual geographical coverage in the five regions reached 100%; whereas therapeutic coverage for the age-eligible population reached 75% for schistosomiases and STHs, and 72% for LF (the total number of individuals that ultimately received drugs was 2,966,000 for LF, among which 1,223,000 school-going age children also received drugs for schistosomiases and STH). The total cost of the campaign was estimated at 1.9 billion MGA (about 2013 US$ 900,000) [24]. **Table 2** in the main text displays a breakdown of costs for the program provided by the government. Program costs included community mobilization and supervision of staff. All drugs were donated by pharmaceutical companies, including Merck, GlaxoSmithKline, Sanofi, and Janssen-Cilag. Similar campaigns were launched by Madagascar’s Ministry of Health in 2014 and in 2015 [16]. Finally, in 2014, the Ministry of Health launched a national “NTD Master Plan” for 2014-2018 to plan the scale-up of national NTD control for schistosomiases, STHs, and LF [6]. In December 2016, the NTD Master Plan was updated to cover the 2016-2020 period [17]. The NTD control campaigns described above follow the strategy recommended for preventive chemotherapy by WHO guidelines, but did not include morbidity management or disability prevention activities (such as clinical treatment for lymphedema or hydrocele resulting from chronic infection).

## **Effectiveness of preventive chemotherapy**

### Cure rates and health outcomes

A range of studies have examined the effect of NTD control programs in real-life settings, including in the context of school-based programs [25-28] (**S7** **Table**). In a longitudinal study in schoolchildren in Uganda, mass treatment with albendazole and praziquantel was associated with a significant decrease in the intensity of *Schistosoma mansoni* infection (37% after one year of treatment) and hookworm infection (53%) [26]. Consistent with these results, a Kenyan longitudinal study found that the cure rate for *S. mansoni* after a single dose of praziquantel was 66% among men between 36 and 82% [29]. A study in the Republic of Congo examined the impact of semiannual mass drug administration (MDA) with albendazole on LF and STHs infection risk. Although the prevalence of LF was reduced by only 10%, the prevalence of hookworm infection in the community was reduced dramatically (91%), although with less strong effects for *Ascaris* and *Trichuris* [30]. For LF, specifically, drug regimens commonly used (such as in WHO’s Global Program to Eliminate LF) are albendazole plus either diethylcarbamazine (DEC) or ivermectin and exhibit excellent microfilaricidal effect, including in studies in sub-Saharan Africa [31, 32]. A study in Kenya found a decrease in prevalence of microfilaraemia corresponding to 45% after one round of MDA [33].

NTD control would generally improve health outcomes. A Tanzanian cross-sectional study, for instance, found that 15 months after school-based deworming with albendazole and praziquantel the prevalence of anemia was reduced by 25% and that of moderate-to-severe anemia (hemoglobin <90 g/l) was reduced by nearly 50% [25]. A longitudinal study in schoolchildren in Uganda, found that mass treatment with albendazole and praziquantel increased hemoglobin concentration after one (0.135g/dL) and two years of treatment (0.303g/dL). Finally, studies have also evaluated different channels of drug delivery to reduce NTD infection. A Nigerian study, for instance, examined different approaches to mass delivery of preventive chemotherapy (praziquantel) to reduce NTD morbidity among school-aged children, including the health facility, school, or community. The study found that the community channel of delivery of praziquantel achieved the best coverage (72% vs. 44% and 29% for the health facilities and school channels, respectively), and included the coverage of *both* in and out-of-school children. One reason for these findings might be that health facility or school attendance may be low.

### Schooling and economic outcomes

NTD control has important epidemiological and socio-economic externalities. Jukes, Drake and Bundy (2008) estimated the global education burden as a result of worm infections at 200 million years of primary schooling lost (assuming a prevalence of 30%, or 169 million infections) based on two studies linking (i) disease to test scores and (ii) test scores to chances of dropping out of school. Evidence from (quasi-)experimental studies show generally positive impacts of deworming treatments on school participation (in the short run) and on academic test scores, employment, and income (in the long run) [34-38]. In a cluster-randomized school-based deworming program in Kenya^[[6]](#footnote-7)^, albendazole twice per year (some schools were additionally treated with praziquantel once per year) and health education (focused on teaching children about avoiding the disease)^[[7]](#footnote-8)^, reduced worm infection prevalence not only among treated individuals but also among untreated individuals attending treatment schools, and individuals attending schools near treatment schools [37, 39]. In the short run, deworming increased school participation^[[8]](#footnote-9)^ in treatment schools by at least 7 percentage points; and contributed to a one-quarter reduction in total school absenteeism. A decade later, average test scores increased by 0.3 standard deviation units (among those ages 0–2 at the time of the program), and educational attainment among girls increased by about 0.30 years [35]. The coefficient on educational attainment in the pooled (both sexes) sample was 0.15 years (standard error=0.14, see Table 2 in Baird et al. 2016). While treatment did not lead to increases in educational attainment among boys, it increased their earnings (e.g., by shifting into jobs that require more work hours and pay better). Moreover, studies that are randomized at the individual level—as opposed to the cluster level that capture possible positive externalities of NTD control—may underestimate the impacts of treatment. (There is, however, some degree of controversy around this evidence. Recent studies suggest that deworming may be less effective [40-42]. A recent, pure replication study found that the total effect on school attendance was reduced from 7.5 percentage points to 3.9 percentage points.) Finally, schooling gains resulting from increased NTD control may have high pecuniary returns in the labor market. In a recent report on comparable estimates of returns to schooling around the world, the returns to one extra year of schooling in Madagascar have been estimated at about 11.1% [43].

### **Healthcare utilization and treatment costs**

LF may lead to lymphedema (swollen limbs and breasts), hydrocele (damage to the genitals), and/or elephantiasis (swollen limbs with thickened, hardened skin) [14]. Approximately 33% of individuals infected with LF suffer from the disabling clinical manifestations of lymphoedema and/or hydrocele [44]. Schistosomiasis produces a wide array of symptoms, including a rash, itchy skin, fever, chills, cough, headache, belly pain, joint pain, and muscle aches [45]. Acute infection with schistosomiasis is characterized by fever (also called “Katayama fever”) in about 50-90% of infected patients among non-immune individuals (typically travelers to endemic areas [46-49]). Chronic infection with schistosomiasis (more common in those living permanently in endemic settings) produces a range of symptoms including abdominal pain and diarrhea. Population surveys have found that diarrhea was reported in 3-55% of infected people, of which 30-60% was attributable to schistosomiasis infections [50-52]. Individuals with STHs may be asymptomatic or experience abdominal pain, blood in stool, loss of appetite, mild nausea, or slow growth. Chronic infection with STHs may lead to, for instance, abdominal pain and diarrhea (ascariasis), iron-deficiency anemia (hookworm disease), as well as abdominal distention and pain (trichuriasis). In children with hookworm disease, the prevalence of hypoferritinemia (ferritin level < 12μg/L) ranged between 33 and 59% depending on the exact type of parasite [53].

Healthcare use in Madagascar is low, possibly due to high transportation costs (e.g., more than 60% of the population lives more than 6 km away from a basic community health center [54]). According to the 2015 Health Statistical Yearbook of Madagascar, 19,781 cases of hydrocele were hospitalized at tertiary hospitals (such as the “Centre Hospitalier de Référence Régionale”); 572 cases of hydrocele were seen at outpatient clinics of tertiary hospitals; and only 766 cases of hydrocele were seen at community outpatient clinics (“Centres de Santé de Base”) [55]. Findings for lymphedema are similar, with only 839 cases seen at community outpatient clinics (“Centres de Santé de Base”) [55]. Given a prevalence of LF of about 4% (all ages), this would translate into roughly 2% of healthcare use on average for the treatment of hydrocele among people affected by LF. In terms of general utilization rates in Madagascar, there were 8,540,000 “total outpatient consultations” and 7,825,000 “new cases”, representing a utilization rate for outpatient consultations of 33% given a population of 24,007,000 in 2015 [55]. There exists strong subnational variation in healthcare usage in Madagascar, with general utilization rates ranging from 10% (Sofia) to 60% (Boeny) depending on the region (**S2 Figure**). Additionally, the 2016 Malaria Indicator Survey (MIS) provides estimates of healthcare utilization for fever among children younger than five years. Treatment was sought at a health facility or community health worker in 60% of children with a fever in the last two weeks.

Data is limited on the cost of treatment for NTD-specific conditions in Madagascar. The cost of LF-related treatment has been estimated at 9,500 MGA (about US$4) for lymphedema and 110,000 MGA (about US$50) for hydrocele in 2016 [56]. These estimates would represent the total cost of care for lymphedema/hydrocele (i.e., without government contribution to payment). Lymphedema and hydrocele (surgical) interventions are typically short outpatient procedures. Drugs are typically not required for morbidity management (as opposed to clinical and/or surgical care) since patients are often not actively infected with the parasite [14]. To our knowledge, the average cost of clinical treatment of conditions specific to schistosomiasis or STHs is not available for Madagascar. The cost of treatment of similar symptoms, however, such as malaria-suspected fever (Paracetamol 500mg 4 tablets/day for 5 days and a single dose of Artemisinin Combined Therapy) has been estimated at 7,360 MGA or about US$2 in 2016 [56]. This estimate corresponds roughly to those of the 2016 MIS for Madagascar on OOP costs for fever treatment [57]. Additionally, the WHO provides country-specific estimates for unit cost values for health care services that can be thought of as “average values of unit costs” [58]. The listed hospital unit costs are specific to public hospitals, with occupancy rate of 80% and representing the “hotel” component of hospital costs, i.e., excluding drugs and diagnostic tests and including other costs such as personnel, capital and food costs. In Madagascar, the cost per outpatient visit at a primary-level hospital was estimated at I$3 (2005 International $ (I$)) or 1,500 Local Currency Units (LCUs) (2005) [59]. The cost per visit at a health center for a 20-minute visit was estimated at I$6 (2005 I$) or 3,100 LCUs (2005) given a population coverage (defined as the percentage of population with physical access to primary health facilities living within 5 km or 1 hour away from the facility) of 50%. These health center costs exclude drugs and diagnostics. The household-level average travel costs per medical visit in Madagascar has been estimated at I$2 (2005 I$) [60]. Finally, 41% of (total) health expenditures would be financed privately in Madagascar according to the World Bank’s World Development Indicators [61].

### **Existing cost-effectiveness and benefit-cost ratio estimates**

Previous studies have estimated the cost-effectiveness of mass school-based treatment programs [62]. The combination of low-cost treatment and high prevalence rates suggests relatively low cost-effectiveness ratios. Indeed, the cost-effectiveness of mass school-based treatment programs for STHs with albendazole or mebendazole was found to be US$2-11 per DALY averted (2008 US$). For schistosomiasis, the cost-effectiveness of treatment with praziquantel and albendazole was US$10-23 per DALY averted; but, with praziquantel alone, was US$410–844. For LF, annual mass drug administration to treat the entire population at risk for 5–7 years with ivermectin and albendazole in Africa to interrupt transmission and achieve elimination was estimated at US$5-10 per DALY averted. LF and onchocerciasis are co-endemic in 28 countries of the WHO African region; both diseases share the same control strategy of preventive chemotherapy to interrupt transmission using ivermectin alone or in combination with albendazole [63]. In Madagascar, however, diethylcarbamazine (DEC) with albendazole is recommended since onchocerciasis is not endemic in the country. In China, treatment with fortiﬁed salt with DEC for LF was highly cost-effective (US$1-4 per DALY averted). The latest *Disease Control Priorities* (DCP) (3^rd^ edition) provides a review of recent cost-effectiveness analyses of preventive chemotherapy, which are generally consistent with earlier estimates. Mass treatment of school-age children in Côte d'Ivoire for STH and schistosomiasis together costs US$114 (US$2012) per DALY averted relative to doing nothing; whereas treatment for LF with albendazole and ivermectin costed US$<4-29 (2012 US$) per DALY averted in South Asia [64].

The prevention of chronic NTDs appears to have large economic benefits. In a study in India, chronic patients with LF gained 11 years of productive life as a result of multiple rounds of MDA, implying average economic benefits of US$449 and a benefit-cost ratio of 53, one of the largest among disease control programs. Estimates from the DCP suggest that the elimination of NTDs would avert a total of I$35 billion OOP health expenditures by affected individuals over 2011-2030 [65, 66]. Progress toward the end of NTDs would avert I$622 billion in wages lost by affected individuals between 2011 and 2030 (excluding longer-term benefits of school attendance for employment). The elimination of NTDs would offer a net benefit to affected individuals of about US$25 for every dollar invested.

## **References for S1 Text**

1. United Nations Development Programme (UNDP). Human Development Report 2016: Human Development For Everyone 2016. Available from: <http://hdr.undp.org/en/countries/profiles/MDG>.

2. The World Bank. Development Indicators 2016. Available from: <http://data.worldbank.org/indicator/NY.GNP.PCAP.CD?locations=MG>.

3. Government of Madagascar. Programme d'Urgences Presidentielles. Plan National de Developpement Interimaire. 2015.

4. You D, Hug L, Ejdemyr S, Idele P, Hogan D, Mathers C, et al. Global, regional, and national levels and trends in under-5 mortality between 1990 and 2015, with scenario-based projections to 2030: a systematic analysis by the UN Inter-agency Group for Child Mortality Estimation. The Lancet. 2015;386(10010):2275-86. doi: 10.1016/s0140-6736(15)00120-8.

5. United Nations Department of Economic and Social Affairs Population Division. World Population Prospects: The 2015 Revision, DVD Edition. 2015.

6. Institute for Health Metrics and Evaluation. Global Health Data Exchange 2017. Available from: <http://ghdx.healthdata.org/gbd-results-tool>.

7. Institute of Health Metrics and Evaluation. Madagascar Country Profile 2016 [14 February 2017]. Available from: <http://www.healthdata.org/madagascar>.

8. UNESCO Institute for Statistics. Education Statistics Madagascar 2016 [14 February 2017]. Available from: <http://data.uis.unesco.org/>.

9. Bashir S. Developing the Workforce, Shaping the Future : Transformation of Madagascar's Post-basic Education. World Bank Working Paper 156. 2009.

10. Glick P, Razafindravonona J, I R. Education and Health Services in Madagascar: Utilization Patterns and Demand Determinants. Cornell University: 2000.

11. United Nations Development Programme (UNDP). The Rise of the South: Human Progress in a Diverse World Human Development Report 2013. 2013.

12. Gakidou E, Cowling K, Lozano R, Murray CJL. Increased educational attainment and its effect on child mortality in 175 countries between 1970 and 2009: a systematic analysis. The Lancet. 2010;376(9745):959-74. doi: 10.1016/s0140-6736(10)61257-3.

13. Cappello M, Hotez PJ, Kamath A. Neglected Tropical Diseases in Sub-Saharan Africa: Review of Their Prevalence, Distribution, and Disease Burden. PLoS Neglected Tropical Diseases. 2009;3(8):e412. doi: 10.1371/journal.pntd.0000412.

14. Centers for Disease Control. Neglected Tropical Diseases 2016 [14 February 2017]. Available from: <https://www.cdc.gov/globalhealth/ntd/>.

15. World Health Organization. Accelerating work to overcome the global impact of neglected tropical diseases – A roadmap for implementation. 2012.

16. Ministère de la Santé Publique, Gouvernement de Madagascar. Rapport synthétique d'activités et de resultats TMM-PAUSENS 2015 Maladies Tropicales Negligées. Magadascar. 2015.

17. Ministère de la Santé Publique, Gouvernement de Madagascar. Direction des Urgences et de Lutte contre les Maladies Endémiques et Négligées: Plan Directeur de Lutte contre les Maladies Tropicales Négligées 2016–2020. Magadascar. 2016.

18. Rasoamanamihaja CF, Rahetilahy AM, Ranjatoarivony B, Dhanani N, Andriamaro L, Andrianarisoa SH, et al. Baseline prevalence and intensity of schistosomiasis at sentinel sites in Madagascar: Informing a national control strategy. Parasites & Vectors. 2016;9(1). doi: 10.1186/s13071-016-1337-4.

19. Institut Pasteur de Madagascar. Mise à jour des données de base sur la prévalence de la Filariose lymphatique, des Schistosomiases et des Géohelminthiases dans les régions Amoron’i Mania, Haute Matsiatra, Androy, Vatovavy Fitovinany et Atsimo Atsinanana. 2015.

20. Kelly H, Cowling BJ. Case Fatality. Epidemiology. 2013;24(4):622-3. doi: 10.1097/EDE.0b013e318296c2b6.

21. World Health Organization. Prevention and Control of Schistosomiasis and Soil-Transmitted Helminthiasis. WHO Technical Series Report 912 Geneva: WHO. 2002.

22. van der Werf MJ, de Vlas SJ, Brooker S, Looman CW, Nagelkerke NJ, Habbema JD, et al. Quantification of clinical morbidity associated with schistosome infection in sub-Saharan Africa. Acta Trop. 2003;86(2-3):125-39. PubMed PMID: 12745133.

23. Crompton D. How Much Human Helminthiasis Is There in the World? The Journal of Parasitology. 1999;85(3):397-403. doi: 10.2307/3285768.

24. Ministère de la Santé Publique, Gouvernement de Madagascar. Rapport synthétique d'activités et de resultats TMM-PAUSENS 2013 Maladies Tropicales Negligées. Magadascar. 2013.

25. Guyatt HL, Brooker S, Kihamia CM, Hall A, Bundy DAP. Evaluation of efficacy of school-based anthelmintic treatments against anaemia in children in the United Republic of Tanzania. B World Health Organ. 2001;79(8):695-703. PubMed PMID: WOS:000170685100003.

26. Kabatereine NB, Brooker S, Koukounari A, Kazibwe F, Tukahebwa EM, Fleming FM, et al. Impact of a national helminth control programme on infection and morbidity in Ugandan schoolchildren. B World Health Organ. 2007;85(2):91-9. PubMed PMID: WOS:000244193900004.

27. Kabatereine NB, Nikolay B, Mwandawiro CS, Kihara JH, Okoyo C, Cano J, et al. Understanding Heterogeneity in the Impact of National Neglected Tropical Disease Control Programmes: Evidence from School-Based Deworming in Kenya. PLOS Neglected Tropical Diseases. 2015;9(9):e0004108. doi: 10.1371/journal.pntd.0004108.

28. Mafe MA, Appelt B, Adewale B, Idowu ET, Akinwale OP, Adeneye AK, et al. Effectiveness of different approaches to mass delivery of praziquantel among school-aged children in rural communities in Nigeria. Acta Tropica. 2005;93(2):181-90. doi: 10.1016/j.actatropica.2004.11.004.

29. Black CL, Steinauer ML, Mwinzi PN, Evan Secor W, Karanja DM, Colley DG. Impact of intense, longitudinal retreatment with praziquantel on cure rates of schistosomiasis mansoni in a cohort of occupationally exposed adults in western Kenya. Trop Med Int Health. 2009;14(4):450-7. doi: 10.1111/j.1365-3156.2009.02234.x. PubMed PMID: 19222824; PubMed Central PMCID: PMCPMC2941893.

30. Pion SDS, Chesnais CB, Bopda J, Louya F, Fischer PU, Majewski AC, et al. The Impact of Two Semiannual Treatments with Albendazole Alone on Lymphatic Filariasis and Soil-Transmitted Helminth Infections: A Community-Based Study in the Republic of Congo. American Journal of Tropical Medicine and Hygiene. 2015;92(5):959-66. doi: 10.4269/ajtmh.14-0661.

31. King JD, Eigege A, Richards F, Jip N, Alphonsus KM, Jiya J, et al. Evidence for Stopping Mass Drug Administration for Lymphatic Filariasis in Some, But Not All Local Government Areas of Plateau and Nasarawa States, Nigeria. The American Journal of Tropical Medicine and Hygiene. 2012;87(2):272-80. doi: 10.4269/ajtmh.2012.11-0718.

32. Lammie PJ, Simonsen PE, Pedersen EM, Rwegoshora RT, Malecela MN, Derua YA, et al. Lymphatic Filariasis Control in Tanzania: Effect of Repeated Mass Drug Administration with Ivermectin and Albendazole on Infection and Transmission. PLoS Neglected Tropical Diseases. 2010;4(6):e696. doi: 10.1371/journal.pntd.0000696.

33. Njenga SM, Wamae CN, Njomo DW, Mwandawiro CS, Molyneux DH. Impact of two rounds of mass treatment with diethylcarbamazine plus albendazole on Wuchereria bancrofti infection and the sensitivity of immunochromatographic test in Malindi, Kenya. Transactions of the Royal Society of Tropical Medicine and Hygiene. 2008;102(10):1017-24. doi: 10.1016/j.trstmh.2008.04.039.

34. Ahuja A, Baird S, Hicks JH, Kremer M, Miguel E, Powers S. When Should Governments Subsidize Health? The Case of Mass Deworming. The World Bank Economic Review. 2015;29(suppl 1):S9-S24. doi: 10.1093/wber/lhv008.

35. Baird S, Hicks JH, Kremer M, Miguel E. Worms at Work: Long-run Impacts of a Child Health Investment. The Quarterly Journal of Economics. 2016;131(4):1637-80. doi: 10.1093/qje/qjw022.

36. Croke K, Hicks JH, Hsu E, Kremer M, Miguel E. Does Mass Deworming Affect Child Nutrition? Meta-analysis, Cost-Effectiveness, and Statistical Power. NBER Working Paper No 22382. 2016. doi: 10.3386/w22382.

37. Miguel E, Kremer M. Worms: Identifying Impacts on Education and Health in the Presence of Treatment Externalities. Econometrica. 2004;72(1):159-217. doi: 10.1111/j.1468-0262.2004.00481.x.

38. Ozier O. Exploiting externalities to estimate the long-term effects of early childhood deworming. Policy Research working paper ; no WPS 7052 Washington, DC: World Bank Group. 2014.

39. Bundy DAP, Wong MS, Lewis LL, Horton J. Control of geohelminths by delivery of targeted chemotherapy through schools. Transactions of the Royal Society of Tropical Medicine and Hygiene. 1990;84(1):115-20. doi: 10.1016/0035-9203(90)90399-y.

40. Davey C, Aiken AM, Hayes RJ, Hargreaves JR. Re-analysis of health and educational impacts of a school-based deworming programme in western Kenya: a statistical replication of a cluster quasi-randomized stepped-wedge trial. International Journal of Epidemiology. 2015;44(5):1581-92. doi: 10.1093/ije/dyv128.

41. Aiken AM, Davey C, Hargreaves JR, Hayes RJ. Re-analysis of health and educational impacts of a school-based deworming programme in western Kenya: a pure replication. International Journal of Epidemiology. 2015;44(5):1572-80. doi: 10.1093/ije/dyv127.

42. Taylor-Robinson DC, Maayan N, Soares-Weiser K, Donegan S, Garner P, Taylor-Robinson DC. Deworming drugs for soil-transmitted intestinal worms in children: effects on nutritional indicators, haemoglobin and school performance. 2012. doi: 10.1002/14651858.CD000371.pub4.

43. Montenegro CE, Patrinos HA. Comparable estimates of returns to schooling around the world. World Bank Policy Research Working Paper. 2014;WPS7020. doi: 10.1596/1813-9450-7020.

44. World Health Organization. Progress report 2000-2009 and strategic plan 2010-2020 of the global programme to eliminate lymphatic filariasis: halfway towards eliminating lymphatic filariasis. Geneva, Switzerland: 2010.

45. Gryseels B, Polman K, Clerinx J, Kestens L. Human schistosomiasis. The Lancet. 2006;368(9541):1106-18. doi: 10.1016/s0140-6736(06)69440-3.

46. Schwartz E, Rozenman J, Perelman M. Pulmonary manifestations of early schistosome infection among nonimmune travelers. Am J Med. 2000;109(9):718-22. PubMed PMID: 11137487.

47. Visser LG, Polderman AM, Stuiver PC. Outbreak of schistosomiasis among travelers returning from Mali, West Africa. Clin Infect Dis. 1995;20(2):280-5. PubMed PMID: 7742430.

48. Centers for Disease Control. Acute schistosomiasis in US travelers returning from Africa. JAMA. 1990;263(16):2165-6. PubMed PMID: 2157076.

49. Colebunders R, Verstraeten T, Van Gompel A, Van den Ende J, De Roo A, Polderman A, et al. Acute Schistosomiasis in Travelers Returning From Mali. J Travel Med. 1995;2(4):235-8. PubMed PMID: 9815399.

50. Booth M, Guyatt HL, Li Y, Tanner M. The morbidity attributable to Schistosoma japonicum infection in 3 villages in Dongting Lake region, Hunan province, PR China. Trop Med Int Health. 1996;1(5):646-54. PubMed PMID: 8911449.

51. Gryseels B. The relevance of schistosomiasis for public health. Trop Med Parasitol. 1989;40(2):134-42. PubMed PMID: 2505372.

52. Guyatt H, Smith T, Gryseels B, Tanner M. Assessing the Public Health Importance of Schistosoma Mansoni in Different Endemic Areas: Attributable Fraction Estimates as an Approach. The American Journal of Tropical Medicine and Hygiene. 1995;53(6):660-7. doi: 10.4269/ajtmh.1995.53.660.

53. Stoltzfus RJ, Chwaya HM, Tielsch JM, Schulze KJ, Albonico M, Savioli L. Epidemiology of iron deficiency anemia in Zanzibari schoolchildren: the importance of hookworms. Am J Clin Nutr. 1997;65(1):153-9. PubMed PMID: 8988928.

54. Ministère de la Santé Publique, Gouvernement de Madagascar. Direction des Urgences et de Lutte contre les Maladies Endémiques et Négligées: Plan Directeur de Lutte contre les Maladies Tropicales Négligées 2014–2018. Magadascar. 2014:1–87.

55. Ministère de la Santé Publique, Gouvernement de Madagascar. Annuaire des Statistiques du Secteur Sante de Madagascar. In: Secretariat General, editor. 2015.

56. World Bank Madagascar (personal communication). 2017.

57. Demographic and Health Surveys. Madagascar MIS, 2016 - MIS Final Report (French). Calverton, MD, USA: 2016.

58. WHO-CHOICE. Choosing Interventions That Are Cost Effective (WHO-CHOICE) Geneva2012. Available from: <http://www.who.int/choice/en/>.

59. World Health Organization. Estimates of Unit Costs for Patient Services for Madagascar 2017. Available from: <http://www.who.int/choice/country/mdg/cost/en/>.

60. Kim S-Y, Sweet S, Slichter D, Goldie SJ. Health and economic impact of rotavirus vaccination in GAVI-eligible countries. BMC Public Health. 2010;10(1). doi: 10.1186/1471-2458-10-253.

61. World Bank. World Bank Indicator: Out-of-pocket health expenditure 2016. Available from: <http://data.worldbank.org/indicator/SH.XPD.OOPC.TO.ZS?locations=MG>.

62. Conteh L, Engels T, Molyneux DH. Socioeconomic aspects of neglected tropical diseases. The Lancet. 2010;375(9710):239-47. doi: 10.1016/s0140-6736(09)61422-7.

63. World Health Organization. Integrating national programmes to eliminate lymphatic filariasis and onchocerciasis. 2015.

64. Fitzpatrick C, Nwankwo U, Lenk E, de Vlas S, Bundy D. An Investment Case for Ending Neglected Tropical Diseases. Disease Control Priorities Network. 3 ed: Oxford University Press; 2017.

65. Gray DJ, Lenk EJ, Redekop WK, Luyendijk M, Rijnsburger AJ, Severens JL. Productivity Loss Related to Neglected Tropical Diseases Eligible for Preventive Chemotherapy: A Systematic Literature Review. PLOS Neglected Tropical Diseases. 2016;10(2):e0004397. doi: 10.1371/journal.pntd.0004397.

66. Redekop WK, Lenk EJ, Luyendijk M, Fitzpatrick C, Niessen L, Stolk WA, et al. The Socioeconomic Benefit to Individuals of Achieving the 2020 Targets for Five Preventive Chemotherapy Neglected Tropical Diseases. PLOS Neglected Tropical Diseases. 2017;11(1):e0005289. doi: 10.1371/journal.pntd.0005289.

1. Gross enrollment ratios include students whose age exceeds the official age group. If there is late enrollment or repetition, the total enrollment can thus exceed the population of the age group that corresponds to the level of education (i.e., >100%). [↑](#footnote-ref-2)
2. Only 0.5% of cases had mixed schistosome infections with *Schistosoma haematobium* and *Schistosoma mansoni*. [↑](#footnote-ref-3)
3. Regions included Amoron’i Mania, Haute Matsiatra, Androy, Vatovavy Fitovinany et Atsimo Atsinanana. [↑](#footnote-ref-4)
4. The drugs distributed during the campaign included Albendazole (400 mg) and Notezine® (100 mg) (diethylcarbamazine) for LF, Praziquantel (600 mg) for schistosomiasis, and Mebendazole (500 mg) for STHs. [↑](#footnote-ref-5)
5. The campaign targeted the age group 5-14, which accounted for 26% of the total population in 2015. The population ages 0-4 was not targeted and accounted for 16% of the total population. (United Nations Population Division, 2016) [↑](#footnote-ref-6)
6. Baseline helminth infection rates were over 90% in study areas (Baird et al. 2016). [↑](#footnote-ref-7)
7. The health education component, however, was suggested to have a minimal impact on behavior (Miguel and Kremer 2004). [↑](#footnote-ref-8)
8. Computed as “pupils present in school during an unannounced NGO visit are considered participants.” [↑](#footnote-ref-9)
