# Supplementary material for: Health, financial, and education gains of investing in preventive chemotherapy for schistosomiasis, soil-transmitted helminthiases, and lymphatic filariasis in Madagascar: A modeling study
Source: PLoS Negl Trop Dis. 2018 Dec 27;12(12):e0007002. doi: 10.1371/journal.pntd.0007002 (PMC6307713; doi:10.1371/journal.pntd.0007002)
Supplement: S1 Table — (DOCX) [file pntd.0007002.s002.docx]

S1 Table. Base-case values and uncertainty ranges used in the probabilistic sensitivity analysis.

| **Parameter** | **Base-case** | **Distribution** | **Distribution parameters** | **Source** |
| --- | --- | --- | --- | --- |
|  |  |  |  |  |
| Prevalence, schistosomiasis (ages 5-14 years) | 25% | Beta | α=26, β=76 | [1] |
| Prevalence, ascariasis (ages 5-14 years) | 26% | Beta | α=27, β=75 | [1] |
| Prevalence, hookworm disease (ages 5-14 years) | 7% | Beta | α=8, β=94 | [1] |
| Prevalence, trichuriasis (ages 5-14 years) | 24% | Beta | α=25, β=77 | [1] |
| Prevalence, lymphatic filariasis (ages 5-14 years) | 3% | Beta | α=4, β=98 | [1] |
| Percentage of infected who are symptomatic, schistosomiasis | 13% | Beta | α=14, β=88 | [2-4] |
| Percentage of infected who are symptomatic, ascariasis | 13% | Beta | α=14, β=88 | [5] |
| Percentage of infected who are symptomatic, hookworm disease | 13% | Beta | α=14, β=88 | [5] |
| Percentage of infected who are symptomatic, trichuriasis | 13% | Beta | α=14, β=88 | [5] |
| Percentage of infected who are symptomatic, lymphatic filariasis | 33% | Beta | α=34, β=68 | [6] |
| Percentage of symptomatic individuals who seek care, schistosomiasis | 60% | Beta | α=61, β=41 | [7] |
| Percentage of symptomatic individuals who seek care, ascariasis | 60% | Beta | α=61, β=41 | [7] |
| Percentage of symptomatic individuals who seek care, hookworm disease | 60% | Beta | α=61, β=41 | [7] |
| Percentage of symptomatic individuals who seek care, trichuriasis | 60% | Beta | α=61, β=41 | [7] |
| Percentage of symptomatic individuals who seek care, lymphatic filariasis | 2% | Beta | α=24, β=978 | [8] |
| Number of DALYs, schistosomiasis (ages 5-14 years) | 15,200 | Normal | μ=15,200, σ=6,530 | [1] |
| Number of DALYs, ascariasis (ages 5-14 years) | 4,709 | Normal | μ=4,709, σ=1,500 | [1] |
| Number of DALYs, hookworm disease (ages 5-14 years) | 2,150 | Normal | μ=2,150, σ=500 | [1] |
| Number of DALYs, trichuriasis (ages 5-14 years) | 3,339 | Normal | μ=3,339, σ=1,100 | [1] |
| Number of DALYs, lymphatic filariasis (ages 5-14 years) | 7,688 | Normal | μ=7,688, σ=2,088 | [1] |
| Effectiveness of chemotherapy, schistosomiasis | 37% | Beta | α=38, β=64 | [9] |
| Effectiveness of chemotherapy, ascariasis | 31% | Beta | α=32, β=70 | [10] |
| Effectiveness of chemotherapy, hookworm disease | 100% | Beta | α=101, β=1 | [10] |
| Effectiveness of chemotherapy, trichuriasis | 13% | Beta | α=14, β=88 | [10] |
| Effectiveness of chemotherapy, lymphatic filariasis | 45% | Beta | α=46, β=56 | [11] |
| Out-of-pocket cost clinical care, schistosomiasis | 1 (2013 USD) | Gamma | Shape: 11, Rate: 11 | [12] |
| Out-of-pocket cost clinical care, ascariasis | 1 (2013 USD) | Gamma | Shape: 11, Rate: 11 | [12] |
| Out-of-pocket cost clinical care, hookworm disease | 1 (2013 USD) | Gamma | Shape: 11, Rate: 11 | [12] |
| Out-of-pocket cost clinical care, trichuriasis | 1 (2013 USD) | Gamma | Shape: 11, Rate: 11 | [12] |
| Out-of-pocket cost clinical care, lymphatic filariasis | 22 (2013 USD) | Gamma | Shape: 50, Rate: 0.22 | [13] |
| School years gained | 0.15 years | Truncated normal | μ=0.15, σ=0.14, lower=0, upper=0.30 | [14] |
|  |  |  |  |  |
| Percentage of workers who hold paid employment jobs | 11% | Beta | α=12, β=90 | [15] |
| Wage returns to one additional year of schooling | 11% | Normal | μ=0.11, σ=0.03 | [16] |
| Average wage | 226 (2013 USD) | Normal | μ=226, σ=100 | [15] |
|  |  |  |  |  |

## **References for S1 Table**

1. Institute for Health Metrics and Evaluation. Global Health Data Exchange 2017. Available from: <http://ghdx.healthdata.org/gbd-results-tool>.

2. Booth M, Guyatt HL, Li Y, Tanner M. The morbidity attributable to Schistosoma japonicum infection in 3 villages in Dongting Lake region, Hunan province, PR China. Trop Med Int Health. 1996;1(5):646-54. PubMed PMID: 8911449.

3. Gryseels B. The relevance of schistosomiasis for public health. Trop Med Parasitol. 1989;40(2):134-42. PubMed PMID: 2505372.

4. Guyatt H, Smith T, Gryseels B, Tanner M. Assessing the Public Health Importance of Schistosoma Mansoni in Different Endemic Areas: Attributable Fraction Estimates as an Approach. The American Journal of Tropical Medicine and Hygiene. 1995;53(6):660-7. doi: 10.4269/ajtmh.1995.53.660.

5. Stoltzfus RJ, Chwaya HM, Tielsch JM, Schulze KJ, Albonico M, Savioli L. Epidemiology of iron deficiency anemia in Zanzibari schoolchildren: the importance of hookworms. Am J Clin Nutr. 1997;65(1):153-9. PubMed PMID: 8988928.

6. World Health Organization. Progress report 2000-2009 and strategic plan 2010-2020 of the global programme to eliminate lymphatic filariasis: halfway towards eliminating lymphatic filariasis. Geneva, Switzerland: 2010.

7. Demographic and Health Surveys. Madagascar MIS, 2016 - MIS Final Report (French). Calverton, MD, USA: 2016.

8. Ministère de la Santé Publique, Gouvernement de Madagascar. Annuaire des Statistiques du Secteur Sante de Madagascar. In: Secretariat General, editor. 2015.

9. Kabatereine NB, Brooker S, Koukounari A, Kazibwe F, Tukahebwa EM, Fleming FM, et al. Impact of a national helminth control programme on infection and morbidity in Ugandan schoolchildren. Bull World Health Organ. 2007;85(2):91-9. PubMed PMID: 17308729; PubMed Central PMCID: PMCPMC2174620.

10. Pion SDS, Chesnais CB, Bopda J, Louya F, Fischer PU, Majewski AC, et al. The Impact of Two Semiannual Treatments with Albendazole Alone on Lymphatic Filariasis and Soil-Transmitted Helminth Infections: A Community-Based Study in the Republic of Congo. American Journal of Tropical Medicine and Hygiene. 2015;92(5):959-66. doi: 10.4269/ajtmh.14-0661.

11. Njenga SM, Wamae CN, Njomo DW, Mwandawiro CS, Molyneux DH. Impact of two rounds of mass treatment with diethylcarbamazine plus albendazole on Wuchereria bancrofti infection and the sensitivity of immunochromatographic test in Malindi, Kenya. Transactions of the Royal Society of Tropical Medicine and Hygiene. 2008;102(10):1017-24. doi: 10.1016/j.trstmh.2008.04.039.

12. World Health Organization. Estimates of Unit Costs for Patient Services for Madagascar 2017. Available from: <http://www.who.int/choice/country/mdg/cost/en/>.

13. World Bank Madagascar (personal communication). 2017.

14. Baird S, Hicks JH, Kremer M, Miguel E. Worms at Work: Long-run Impacts of a Child Health Investment. The Quarterly Journal of Economics. 2016;131(4):1637-80. doi: 10.1093/qje/qjw022.

15. Institut National de la Statistique. Enquête nationale sur l’emploi et secteur informel (ENEMPSI). Government of Madagascar, 2012.

16. Montenegro CE, Patrinos HA. Comparable estimates of returns to schooling around the world. World Bank Policy Research Working Paper. 2014;WPS7020. doi: 10.1596/1813-9450-7020.
