# Supplementary material for: Health, financial, and education gains of investing in preventive chemotherapy for schistosomiasis, soil-transmitted helminthiases, and lymphatic filariasis in Madagascar: A modeling study
Source: PLoS Negl Trop Dis. 2018 Dec 27;12(12):e0007002. doi: 10.1371/journal.pntd.0007002 (PMC6307713; doi:10.1371/journal.pntd.0007002)
Supplement: S2 Table — (DOCX) [file pntd.0007002.s003.docx]

## S2 Table. Estimated unit cost of drugs (per tablet) for neglected tropical diseases (2012).

*Notes:* In our base-case analysis, we assumed that drugs were donated by pharmaceutical companies. We therefore used unit costs of drugs to assess the sensitivity of our results. Source: World Bank 2017 (personal communication); World Health Organization.
