# Supplementary material for: Health, financial, and education gains of investing in preventive chemotherapy for schistosomiasis, soil-transmitted helminthiases, and lymphatic filariasis in Madagascar: A modeling study
Source: PLoS Negl Trop Dis. 2018 Dec 27;12(12):e0007002. doi: 10.1371/journal.pntd.0007002 (PMC6307713; doi:10.1371/journal.pntd.0007002)
Supplement: S3 Table — (DOCX) [file pntd.0007002.s004.docx]

## S3 Table. Subnational analysis: cost-effectiveness of neglected tropical disease control by different district prevalence categories in Madagascar.

*Notes:* We used 5%, 25%, 45%, 65%, and 85% as the district prevalence of all five NTDs. For instance, in the districts Ampanihy, Beloha, Ihosy and Toliara II in South-Western Madagascar, the prevalence of STHs ranged from 1 to 8%. Conversely, in the districts Mananara Avaratra, Maroantsetra, Toamasina I and Toamasina II in North-Eastern Madagascar, the prevalence of STHs ranged from 77 to 99%. We assumed an average district target population of 63,250 school-aged children ages 5-14 years old (calculated as the total school-going age population of Madagascar divided by 100 since there are approximately 100 districts in Madagascar). Household expenditures are reported in USD 2013.
