# Supplementary material for: Health, financial, and education gains of investing in preventive chemotherapy for schistosomiasis, soil-transmitted helminthiases, and lymphatic filariasis in Madagascar: A modeling study
Source: PLoS Negl Trop Dis. 2018 Dec 27;12(12):e0007002. doi: 10.1371/journal.pntd.0007002 (PMC6307713; doi:10.1371/journal.pntd.0007002)
Supplement: S4 Table — Each NTD intervention was modelled independently with its own programmatic cost. (DOCX) [file pntd.0007002.s005.docx]

## S4 Table. Benefit-cost analysis of neglected tropical disease (NTD) control in Madagascar. Each NTD intervention was modelled independently with its own programmatic cost.

*Notes:* We used the total cost of the 2013 campaign targeting school-going age children as programmatic cost for each NTD (1,244,762 2013 USD), and 1 DALY = 1,000 USD for the economic gains associated with one DALY averted in low-resource settings [1]. Monetary gains of schooling used data on wages from the Labor Force Survey of Madagascar (2012) [2]. We discounted wage benefits at 3% per year over 20 years [3].

## **References for S4 Table**

1. Jamison DT, Prabhat J, Laxminarayan R, Ord T. Copenhagen Consensus Challenge Paper: Infectious disease, injury, and reproductive health. Copenhagen: Copenhagen Consensus Center, 2012.

2. Institut National de la Statistique. Enquête nationale sur l’emploi et secteur informel (ENEMPSI). Government of Madagascar, 2012.

3. Drummond M. Methods for the economic evaluation of health care programmes. Fourth edition. ed. Oxford, United Kingdom ; New York, NY, USA: Oxford University Press; 2015. xiii, 445 pages p.
