# Supplementary material for: Health, financial, and education gains of investing in preventive chemotherapy for schistosomiasis, soil-transmitted helminthiases, and lymphatic filariasis in Madagascar: A modeling study
Source: PLoS Negl Trop Dis. 2018 Dec 27;12(12):e0007002. doi: 10.1371/journal.pntd.0007002 (PMC6307713; doi:10.1371/journal.pntd.0007002)
Supplement: S5 Table — (DOCX) [file pntd.0007002.s006.docx]

## S5 Table. Univariate sensitivity analysis: drugs used for preventive chemotherapy for neglected tropical diseases in Madagascar are not donated for free by pharmaceutical companies.

*Notes:* We included unit cost of drugs (per tablet) for neglected tropical diseases in addition to costs related to the roll-out of the program (e.g., training, campaigns).
