# Supplementary material for: Health, financial, and education gains of investing in preventive chemotherapy for schistosomiasis, soil-transmitted helminthiases, and lymphatic filariasis in Madagascar: A modeling study
Source: PLoS Negl Trop Dis. 2018 Dec 27;12(12):e0007002. doi: 10.1371/journal.pntd.0007002 (PMC6307713; doi:10.1371/journal.pntd.0007002)
Supplement: S6 Table — (DOCX) [file pntd.0007002.s007.docx]

## S6 Table. Benefit-cost analysis of neglected tropical disease control in Madagascar using an alternative benefit scenario: assuming all infections occur within the same individuals (100% overlap).

*Notes:* We assumed 100% co-infection of NTDs (i.e., all NTD infections occur within the same individuals). We used the highest prevalence among NTDs in our study which was 26% (ascariasis). We then hypothesized that all other NTD infections (schistosomiasis, other STHs, LF) occurred exclusively in those individuals. We also assumed the lowest possible effectiveness of preventive chemotherapy (13%) since in (potentially) co-infected individuals *all* infections would need to be prevented for them to be able to participate in school (i.e., preventing only one NTD in co-infected individuals has little bearing on their possible health, wealth, or schooling gained). See references for the economic gains associated with one DALY in lower-resource settings [1]. We estimated the monetary gains of schooling using data on wages from the Labor Force Survey of Madagascar (2012) [2]. We counted wage benefits over 20 years and discounted at 3% per year.

**References for S6 Table**

1. Jamison DT, Prabhat J, Laxminarayan R, Ord T. Copenhagen Consensus Challenge Paper: Infectious disease, injury, and reproductive health. Copenhagen: Copenhagen Consensus Center, 2012.

2. Institut National de la Statistique. Enquête nationale sur l’emploi et secteur informel (ENEMPSI). Government of Madagascar, 2012.
