# Supplementary material for: Health, financial, and education gains of investing in preventive chemotherapy for schistosomiasis, soil-transmitted helminthiases, and lymphatic filariasis in Madagascar: A modeling study
Source: PLoS Negl Trop Dis. 2018 Dec 27;12(12):e0007002. doi: 10.1371/journal.pntd.0007002 (PMC6307713; doi:10.1371/journal.pntd.0007002)
Supplement: S7 Table — (DOCX) [file pntd.0007002.s008.docx]

## S7 Table. Cure rates of selected drugs for control of neglected tropical diseases.

*Assessed the impact of a mass drug administration campaign.
