# Supplementary material for: Health, financial, and education gains of investing in preventive chemotherapy for schistosomiasis, soil-transmitted helminthiases, and lymphatic filariasis in Madagascar: A modeling study
Source: PLoS Negl Trop Dis. 2018 Dec 27;12(12):e0007002. doi: 10.1371/journal.pntd.0007002 (PMC6307713; doi:10.1371/journal.pntd.0007002)
Supplement: S1 Fig — (DOCX) [file pntd.0007002.s009.docx]

## S1 Figure. Prevalence of soil-transmitted helminthiases (STH) by district in Madagascar (2016).

**
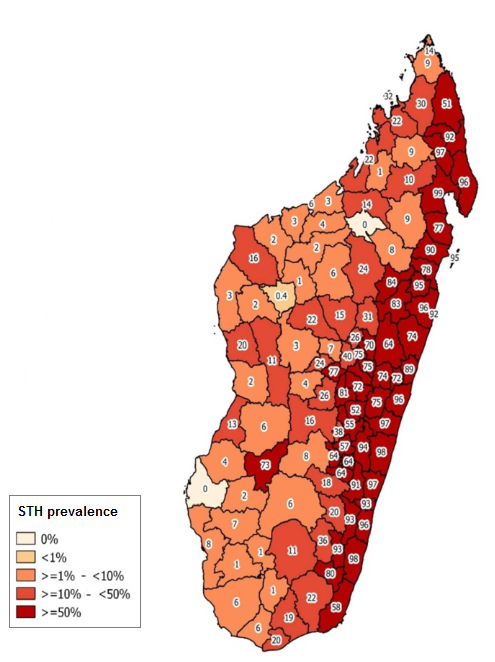
**

*Notes:* Source: Ministère de la Santé Publique. Direction des Urgences et de Lutte contre les Maladies Endémiques et Négligées: Plan Directeur de Lutte contre les Maladies Tropicales Négligées 2016–2020. Magadascar. 2016.
