# Supplementary material for: Health, financial, and education gains of investing in preventive chemotherapy for schistosomiasis, soil-transmitted helminthiases, and lymphatic filariasis in Madagascar: A modeling study
Source: PLoS Negl Trop Dis. 2018 Dec 27;12(12):e0007002. doi: 10.1371/journal.pntd.0007002 (PMC6307713; doi:10.1371/journal.pntd.0007002)
Supplement: S2 Fig — (DOCX) [file pntd.0007002.s010.docx]

## S2 Figure. Distribution of outpatient visits at primary care centers (“Centres de Santé de Base”) by region in Madagascar (2015).


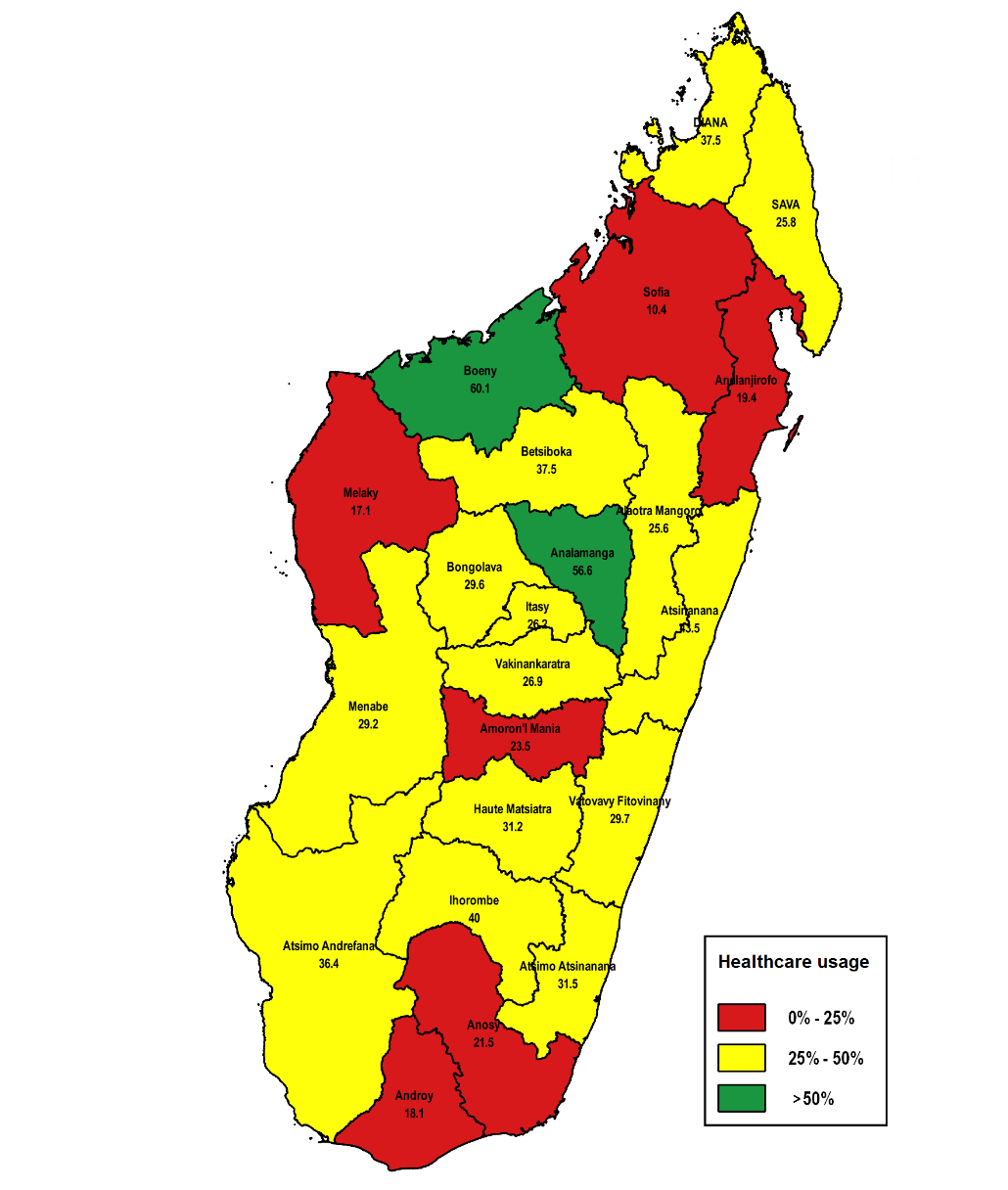


*Notes:* Source: Ministère de la Santé Publique. Annuaire des Statistiques du Secteur Santé de Madagascar. 2015.
